# Repression of Meiotic Genes by Antisense Transcription and by Fkh2 Transcription Factor in *Schizosaccharomyces pombe*

**DOI:** 10.1371/journal.pone.0029917

**Published:** 2012-01-06

**Authors:** Huei-Mei Chen, Adam P. Rosebrock, Sohail R. Khan, Bruce Futcher, Janet K. Leatherwood

**Affiliations:** Department of Molecular Genetics and Microbiology, Stony Brook University, Stony Brook, New York, United States of America; University of Kent, United Kingdom

## Abstract

In *S. pombe*, about 5% of genes are meiosis-specific and accumulate little or no mRNA during vegetative growth. Here we use Affymetrix tiling arrays to characterize transcripts in vegetative and meiotic cells. In vegetative cells, many meiotic genes, especially those induced in mid-meiosis, have abundant antisense transcripts. Disruption of the antisense transcription of three of these mid-meiotic genes allowed vegetative sense transcription. These results suggest that antisense transcription represses sense transcription of meiotic genes in vegetative cells. Although the mechanism(s) of antisense mediated transcription repression need to be further explored, our data indicates that RNAi machinery is not required for repression. Previously, we and others used non-strand specific methods to study splicing regulation of meiotic genes and concluded that 28 mid-meiotic genes are spliced only in meiosis. We now demonstrate that the “unspliced” signal in vegetative cells comes from the antisense RNA, not from unspliced sense RNA, and we argue against the idea that splicing regulates these mid-meiotic genes. Most of these mid-meiotic genes are induced in mid-meiosis by the forkhead transcription factor Mei4. Interestingly, deletion of a different forkhead transcription factor, Fkh2, allows low levels of sense expression of some mid-meiotic genes in vegetative cells. We propose that vegetative expression of mid-meiotic genes is repressed at least two independent ways: antisense transcription and Fkh2 repression.

## Introduction

In a simple case of gene expression only the sense DNA strand of a protein-coding region is transcribed. In recent years it has become apparent that in many cases from yeasts to mammals the antisense strand is also transcribed [1]–[5]. The fission yeast *Schizosaccharomyces pombe* also makes antisense RNAs that are antisense to coding regions. Depending on the experimental methods and arbitrary cut-off thresholds for length and intensity of antisense RNA, the number ranges from 37 to over 2000 [6]–[9].

Antisense transcripts could be irrelevant by-products of some aspect of the transcriptional machinery; alternatively, they could play some regulatory role, possibly through RNA interference, or through transcription interference. A few antisense RNAs over differentially expressed genes have been shown to play regulatory roles in *S. cerevisiae*. In vegetative cells, transcription of the key meiotic regulator *IME4* is repressed by an antisense RNA, which spans the entire length of *IME4* [10], [11]. The repression mechanism is most consistent with transcription interference, in which transcription of the antisense strand suppresses transcription on the sense strand [10], [11]. Long antisense RNAs were also found for *PHO84* [12]. Transcription of *PHO84* is regulated by phosphate level [13]. This antisense RNA recruits histone deacetylase to the promoter region of *PHO84*, deacetylates the histones and represses sense transcription [12]. *S. cerevisiae* does not have RNAi machinery, while *S. pombe* does. In organisms with RNAi machinery, long non-coding RNAs have the potential to be processed into small RNAs and repress target gene expression [14], [15].

In this report we use Affymetrix tiling arrays to characterize *S. pombe* transcripts in vegetative and meiotic cells in detail. In vegetative cells, we identified a group of 116 genes that have more antisense RNAs than sense RNAs. A large proportion of these antisense RNAs lie over genes that are induced during meiosis, especially mid-meiotic genes. Several previous studies are also consistent with the idea that meiotic genes have high levels of antisense transcripts in vegetative cells [7]–[9]. Here, we show that these antisense transcripts are important for maintaining tight vegetative repression of mid-meiotic genes.

The high levels of vegetative antisense transcripts over meiotic genes have another consequence. Previous studies have concluded that 28 mid-meiotic genes are regulated by meiosis-specific splicing, because their introns are not spliced out in vegetative cells, but then become spliced during mid-meiosis [7], [16]–[20]. However, the splicing assay used in these reports was not strand-specific. Vegetative antisense transcripts, whose presence was not known at the time of these previous studies, can mimic the appearance of unspliced sense RNAs in the assays that were used. That is, it is possible that the unspliced transcripts of meiotic genes seen in vegetative cells in previous studies were actually (inherently unspliceable) antisense transcripts, while the spliced transcripts seen in meiotic cells resulted not from a change in splicing, but simply from expression of the sense strand. We have already published preliminary evidence supporting this possibility [9]. Here, we provide further evidence that many of the mid-meiotic genes previously thought to be regulated at the level of splicing are not regulated in that way, but instead are potential targets of antisense-mediated regulation.

Mei4, the meiosis-specific forkhead transcription factor, transcriptionally induces these mid-meiotic genes. Interestingly, another forkhead transcription factor, Fkh2, is proposed to be the key repressor of splicing for 11 mid-meiotic genes in vegetative cells [18]. We found 10 of these genes have predominant antisense RNAs in vegetative cells. Therefore, we investigated whether Fkh2 is instead a key player in the balance between sense and antisense transcription. Finally, detailed characterized of transcription shows a number of unique solutions to regulatory problems in the transition to meiosis.

## Results

### Characterization of *S. pombe* transcripts

To achieve a detailed characterization of transcripts and splicing in *S. pombe*, we used Affymertix *S. pombe* 1.0 tiling arrays to analyze transcripts isolated from vegetative and meiotic cells. Such arrays have 25-nucleotide oligos tiling the entire genome on both DNA strands. RNA for analysis was converted to cDNA by priming with an anchored oligo dT primer, and extending the primer with reverse transcriptase (Materials and Methods). cDNAs were then size-selected to remove fragments smaller than 70 nucleotides. The cDNAs obtained in this way represent long polyadenylated RNA species. Because reverse transcriptases can use either RNA or DNA as a template, spurious second-strand cDNA can be made from first strand cDNA during the reverse transcription reaction [21], [22]. On the strand specific tiling array, such second-strand cDNAs would appear as spurious antisense transcripts. To prevent synthesis of second-strand cDNAs, actinomycin D (Act D) was added to the reverse transcription reaction. Act D inhibits use of DNA templates by reverse transcriptase, and so reduces second-strand cDNA synthesis without affecting first-strand cDNA synthesis [23].

The first-strand cDNAs were hybridized to the Affymetrix *S. pombe* 1.0 arrays. The hybridization signals were normalized and partitioned into segments with constant probe hybridization intensities (see Materials and Methods) as a way of defining transcripts. Full results are available at ArrayExpress under accession number: E-MEXP-3414. These results contain a wealth of detailed information about the transcripts in vegetative and meiotic cells; because of technical details of the method, some of this information is not apparent in previously published studies (Table S1). Here, we will focus on the results pertaining to antisense transcripts, splicing, and the regulation of meiosis.

Because we were interested in the issue of meiosis-specific splicing, we examined genes previously identified as expressed and unspliced in vegetative cells, but spliced in meiotic cells [7], [16]–[20]. However, strikingly, in many of these cases, the vegetative cells expressed little or no detectable sense transcript, spliced or unspliced, but instead expressed anti-sense transcripts. This finding casts doubt on the original observation of meiosis-specific splicing for these genes (see below).

**Figure 1:**
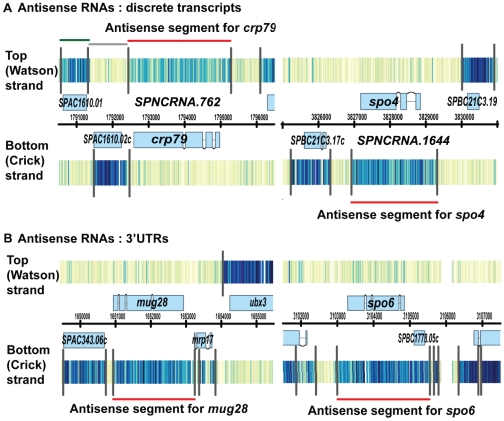
Example of high-resolution tiling array data. (A) Genes drawn above the black chromosome co-ordinates line are encoded on the top (Watson) strand (5′ to 3′ is left to right), while genes drawn below the line are encoded on the bottom (Crick) strand (5′ to 3′ is right to left). Exons are shown as light blue boxes. Introns are shown as thin lines linking the exons. Signal intensity for each probe is shown as a vertical line with color ranging from light yellow (low signal) to dark blue (high signal). Black vertical lines are algorithmically calculated boundaries separating two regions of different probe intensities. A segment is the region between two boundary lines. Segments fall mainly into three groups: sense segments, antisense segments, and non-annotated segments. Some segments are color-coded for demonstration. The green segment (top left) is the sense segment for SPAC1610.01. The adjacent grey segment is the antisense segment for SPAC2610.02c, but since the signal intensity is low, there is no apparent antisense RNA. The red segment is the antisense segment for *crp79^+^,* and the relatively high signal intensity implies an antisense RNA. In panel (A), the antisense RNAs of *spo4^+^* and *crp79^+^* (red lines) are discrete transcript units that do not connect with other features. The antisense transcripts for *crp79^+^* and *spo4^+^* were previously annotated as SPNCRNA.762 and SPNCRNA1664, respectively. In panel (B), the antisense RNAs of *spo6^+^* and *mug28^+^* (red lines) are the 3′ UTRs of the adjacent genes SPBC1778.05c and *mrp17^+^,* respectively. These 3′ UTRs are unusually long.

Because of the striking antisense transcripts seen over genes of interest to us, we characterized antisense transcripts genome-wide. We restricted our attention to antisense segments with signal intensity above a threshold (Materials and Methods). These antisense RNAs fell into two major groups. One group was composed of apparently discrete transcription units (Figure 1A, antisense RNAs for *crp79^+^* and *spo4^+^*). The antisense RNA of *crp79^+^* seems to originate from the bi-directional promoter of SPAC1610.02c; such antisense transcripts from a bi-directional promoter are apparently fairly common. The second group appeared to be a continuous extension of a 3′UTR of an annotated gene on the opposite strand (Figure 1B, antisense RNAs for *mug28^+^* and *spo6^+^*), although it was possible that the antisense RNA was an independent transcript starting very close to the end of the 3′ UTR. To distinguish these possibilities, we used Northern blotting to determine the length of antisense RNAs. For all four antisense RNAs tested, the length of the antisense RNA matches the length deduced from the tiling array, indicating that these antisense RNAs are indeed long 3′UTRs. These Northern results were previously published [9]. Many of these 3′UTR antisense RNAs are extremely long, several kilobases in some cases. Most antisense RNAs from both groups covered the entire coding region of the sense gene (e.g., Figure 1). In total, we identified 1540 long polyadenylated antisense RNAs; that is, nearly 31% of protein-coding genes have antisense transcripts.

### The antisense RNAs in vegetative cells are preferentially found over mid-meiotic genes

**Figure 2:**
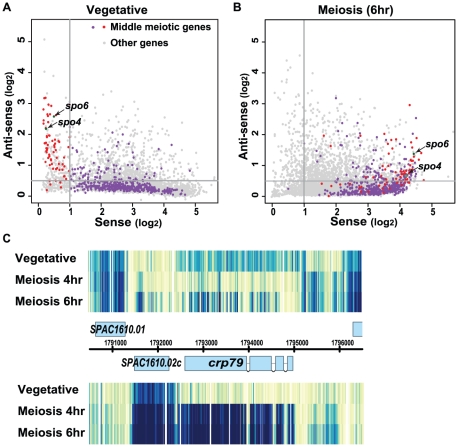
Middle meiosis-specific genes are associated with antisense RNA in vegetative cells. Each dot represents one gene; the x-axis and y-axis indicates the sense and antisense RNA level, respectively. Previously identified Mei4-dependent middle meiotic genes are shown as purple or red dots. Purple genes had sense RNA levels above 1 in vegetative cells, while red genes had very low sense RNA levels. Two meiotic genes, *spo4^+^* and *spo6^+^,* studied in detail in this work are labeled (green dots). Other genes are shown as grey dots. (A) Asynchronous vegetative cells. Sense expression levels were distributed across a wide range. Antisense RNA levels were generally much lower than the sense level for a given gene. Mei4-dependent middle meiotic genes were over-represented in the group that had high antisense (>0.5) and low sense (<1) RNA (red dots). (B) Middle meiotic cells (6 hr). At middle meiosis, these middle meiotic genes were highly induced (the purple and red dots shifted toward the right). The antisense RNA level for meiosis-specific middle genes had a decreased level (as the red dots shifted toward the bottom). (C) Behavior of sense and antisense RNAs of *crp79^+^*. In vegetative cells, *crp79^+^* has abundant antisense RNA (top) but no sense RNA (bottom). At 4 hr meiosis, the antisense largely decreases and sense RNA appears. At 6 hr meiosis, the antisense RNA reappears with different transcript boundaries.

To help understand the possible significance of the antisense transcripts, we calculated the abundance of sense transcript and antisense transcript for each gene (Materials and Methods). These values for every *S. pombe* gene are presented in Figure 2A (vegetative cells) and Figure 2B (meiotic cells, 6 hr after meiotic induction) as a dot plot in which each dot represents one gene, the x-axis represents the sense signal intensity, and the y-axis represents the antisense signal intensity. The numerical values are presented in Table S2.

The majority of genes have much stronger sense signals than antisense signals. However, in vegetative cells, there is a small set of genes with relatively low sense signals, and relatively high antisense signals (i.e., a relatively high ratio of antisense transcript to sense transcript). We focused on the genes in the upper left rectangle of Figure 2A; i.e., the genes with a sense intensity of less than 1, and an antisense intensity of more than 0.5. (Genome-wide, the median intensity of the sense transcripts is 2.98, and the median intensity of anti-sense transcripts is 0.28.) There are 116 genes in this group. GO term analysis [24] of these genes found a striking enrichment of meiotic genes (30%, p<9×10^−11^), especially genes annotated as meiotic M phase (mid-meiosis) (Table S4). Although the other 70% of the genes were not annotated as meiotic genes, most of them were also induced during meiosis (Figure S1). Only 10 of the 116 genes do not increase during meiosis. We conclude that in vegetative cells the genes that have a high ratio of antisense RNA to sense RNA are strongly enriched for meiotic genes, particularly those expressed in mid-meiosis.

We excluded those genes that have high antisense (>0.5) and also high sense (>1) levels from the GO term analysis for two reasons. First, it has been shown that increased chromatin accessibility or high local concentrations of transcription apparatus due to sense RNA transcription permits transcription of spurious antisense RNA [6]. Second, Rdp1, the RNA dependent RNA polymerase, can synthesize antisense RNA using the sense RNA as template [25]. Consistent with this, we found that some antisense RNAs decreased in the *rdp1Δ* strain (data not shown).

### Antisense RNAs of mid-meiotic genes decrease during meiosis

Antisense transcripts change both in abundance and also in transcript boundaries in meiosis. Furthermore, when transcripts drop dramatically in abundance, it becomes difficult to assign boundaries. For example, the antisense RNA of *crp79^+^* is reduced at 4 hr of meiosis. Antisense transcript re-appears at 6 hr, but with different transcript boundaries (Figure 2C). Because the boundaries of antisense RNAs often change or cannot reliably be assigned due to lowered expression level during some portion of meiosis, it is challenging to track which antisense RNA in vegetative cells corresponds to which antisense RNA in meiosis. Therefore, to present the changes of sense and antisense RNA levels during meiosis we used the average probe intensity method (Materials and Methods; Figure 2A and 2B). Overall, we observed a negative genome-wide correlation (−0.221) between the abundance of sense and antisense RNAs. This suggests that sense and antisense transcription are, to some extent, mutually suppressive. Because sense transcription and antisense transcription compete for a common DNA template, transcription interference is one possible reason for this mutual suppression (see Discussion).

About 500 genes are induced 4-fold or more during mid-meiosis and are classified as mid-meiotic genes [26]. Mei4, the meiotic forkhead transcription factor, is essential for induction of almost all of these mid-meiotic genes [27]. In the dot plots (Figure 2A and 2B) all the Mei4-induced mid-meiotic genes are colored purple or red (*spo4^+^* and *spo6^+^*, also induced by Mei4, are colored green). The purple genes are induced in meiosis, but also have substantial expression (sense RNA >1) in vegetative cells. This purple group contains genes for chromosome segregation, including mitotic activators, the condensin complex, the spindle pole body complex and mitotic kinases [27]. These genes are used for both mitotic and meiotic nuclear divisions. In contrast, the red genes have little or no expression in vegetative cells. The red genes constitute the “meiosis-specific” genes, such as s*po4^+^* and *spo6^+^*, the meiotic Cdc7-like kinase and its regulatory factor [28], and *mug28^+^* and *crp79* ^+^ [29], two meiotic RNA-binding proteins. It is apparent (Figure 2A and 2B) that these meiosis-specific mid-meiotic genes—the red genes—have the most abundant antisense transcripts in vegetative cells. For these meiosis-specific genes, the negative correlation between sense and antisense RNAs is more pronounced (−0.453, Figure S2).

### Disruption of antisense transcription allows sense RNA expression

**Figure 3:**
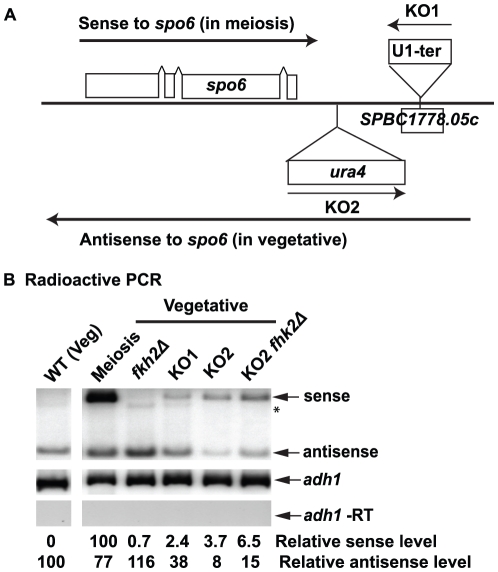
Disruption of antisense transcription allows *spo6^+^* sense transcription in vegetative cells. (A) Illustration of *spo6* antisense disruption strains. The arrow above *spo6^+^* represents the sense RNA. The arrow below *spo6^+^* represents the antisense RNA, which is the long 3′UTR of SPBC1178.05c (see Figure 1B). Two antisense knockout strains were constructed. KO1: insertion of the U1 terminator into SPBC1778.05c in the same transcription direction as *SPBC1778.05c*. KO2: insertion of the *ura4^+^* cassette (promoter-*ura4^+^*-terminator) between *spo6^+^* and SPBC1778.05c in the same transcription direction as s*po6^+^*. (B) Radioactive PCR detection of *spo6^+^* sense and antisense RNAs. *adh1^+^* is included as internal loading control, and *adh1^+^* (-RT) indicates no genomic DNA contamination. Antisense RNAs decrease and sense RNAs appear in both antisense KO strains. Fkh2 has minimal effect on *spo6* sense and antisense transcription. The relative sense and antisense RNA level were quantified and normalized to *adh1^+^* to show the fold change. The *adh1* transcript drops by about 30% in meiosis, and this was taken into account in the normalization. Two or more isolates for each strain were assayed. This figure shows the representative result.

Given that many meiosis-specific genes have high levels of antisense transcripts in vegetative cells and given the negative correlation between sense and antisense RNA abundance, we wanted to know if antisense transcription inhibits sense transcription of the mid-meiotic genes. To test this, we disrupted antisense transcription by insertion of a transcription terminator derived from *ura4^+^* [30]. Insertion of the U1 terminator will disrupt antisense transcription only if the antisense RNA seen on the tiling array is a continuous RNA (rather than small fragments of RNAs) and is transcribed by RNA polymerase II. We and collaborators used Northern blot analysis to show that the antisense RNAs for *spo6^+^*, *spo4^+^, mug28^+^* and other mid-meiotic genes are indeed long RNAs with their sizes corresponding to our tiling array results [9]. Partly because the transcription start and stop sites of the *spo6^+^* antisense RNA had previously been assigned by 5′ and 3′RACE [31], we started the antisense disruption experiment with *spo6^+^*. We inserted the U1 terminator into SPBC1778.05c, the gene adjacent to *spo6^+^* and the source of antisense RNA for *spo6^+^* (see Figure 1B for tiling array data and Figure 3A for strain construction). This strain was named *spo6*-AS-KO1, or KO1 in short. Using radioactive PCR, we analyzed the *spo6^+^* sense and antisense RNA level from WT vegetative cells, meiotic cells and the KO1 vegetative cells (Figure 3B). A decreased antisense RNA level was observed in the KO1 strain, indicating that the U1 terminator successfully blocked some antisense transcription. Whereas no sense RNA could be seen for *spo6^+^* in wild-type vegetative cells, a sense *spo6^+^* RNA was seen in the KO1 vegetative cells. However, this *spo6^+^* sense transcript in the KO1 strain was much less abundant than the transcript seen in meiosis. This suggests that the maximum *spo6* sense RNA expression depends on meiosis-specific transcription induction, and that the antisense RNA in vegetative cells may act to prevent low-level basal expression of *spo6*.

A potential complication is that insertion of the U1 terminator might change the chromatin structure downstream of *spo6^+^* and somehow alter the expression of *spo6^+^.* Therefore, a second method was used to inhibit antisense transcription. A *ura4^+^* cassette (promoter-*ura4^+^*-terminator) was inserted downstream of *spo6^+^* such that *ura4^+^* transcription was convergent with the antisense transcription, generating strain *spo6-*AS-KO2. The intent is that *ura4^+^* transcription should decrease antisense transcription by transcription interference. This KO2 strain also successfully decreased antisense transcription (Figure 3B). As in the KO1 strain, the level of sense RNA increased in the KO2 strain compared to the wild-type strain. Some antisense transcription remained in both KO1 and KO2 strains. It is possible that the U1 terminator did not terminate efficiently in the KO1 context and/or that cryptic transcription initiation sites were activated in these constructs. The same two antisense disruption strategies were used to block antisense transcription for two other middle meiotic genes, *spo4^+^* and *mug28^+^* (Figure S4). For both genes, the antisense RNA decreased in the AS-KO strains, and in these strains, a low level of sense RNA appeared. Based on these results for three genes, we conclude that these antisense RNAs prevent basal level sense transcription of mid-meiotic genes in vegetative cells.

We note that in the radioactive PCR assay of Figure 3, the level of *spo6* antisense does not drop as much in meiosis as in the tiling array assay of Figure 2. This is possibly because the antisense transcript is degraded into semi-stable fragments in meiosis, and the different assays capture different aspects of this behavior (the primers for the radioactive PCR capture one of the most stable fragments of the transcript. Figure S3). In addition, the assays are necessarily normalized in different ways.

### The RNAi pathway and heterochromatin formation are not involved in antisense-mediated repression

**Figure 4:**
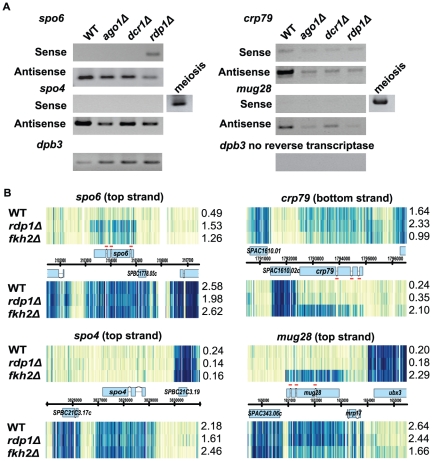
RNAi machinery is not involved in antisense-mediated repression. (A) Testing the sense and antisense RNA level in the RNAi mutants, a*go1Δ*, *dcr1Δ* and *rdp1Δ,* using radioactive PCR. These mutants did not affect the sense RNA level for *spo4^+^*, *crp79^+^* and *mug28^+^*. Only *spo6^+^* sense RNA was slightly induced in the *rdp1Δ* strain. The *dpb3^+^* level indicates equal loading and no reverse transcriptase indicates that samples were not contaminated with genomic DNA. Two isolates for each strain were assayed. This figure shows the representative result. (B) Tiling array data confirmed that *spo6^+^* was induced in the *rdp1Δ* strain (left panel). *spo4^+^*, *crp79^+^* and *mug28^+^* were not affected by *rdp1^−^* (right panel). In the *fkh2Δ mei4Δ* strain (shown as *fkh2Δ*), the sense RNA for *spo6^+^* was only slightly elevated, whereas *crp79^+^* and *mug28^+^* sense RNA were clearly induced. Note that in every condition when sense transcripts for *spo6^+^, crp79^+^* and *mug28^+^* are detectable, the intron probes (red lines) have lower intensities than exon probes, indicating that splicing occurred in vegetative cells. The sense and antisense average intensities are presented on the right of each panel.

We next investigated the molecular mechanism of antisense-mediated repression. One possibility is that these antisense RNAs might be processed into small RNAs by the RNAi pathway. Subsequently, the small RNAs might repress sense RNAs by directly interacting with sense RNA and/or by inducing heterochromatin formation (reviewed in [32]). To assess involvement of the RNAi pathway, we assayed the sense and antisense RNAs of four mid-meiotic genes (*spo4^+^*, *spo6^+^*, *mug28^+^* and *crp79^+^*) in three mutants that affect the RNAi pathway. These mutants were *ago1Δ* (Argonaute), *dcr1Δ* (Dicer) and *rdp1Δ* (RNA-dependent RNA polymerase). The *spo6^+^* sense RNA was somewhat elevated in the *rdp1Δ* strain (Figure 4A and B), but otherwise, we could not detect any elevation of sense transcript in any of the other mutants. This is consistent with the idea that the general RNAi pathway is not the main mechanism for this antisense-mediated repression. Also consistent with this view, we and collaborators previously showed using transcript sequencing that sense/antisense ratios were not affected for *spo4*, *spo6*, *mug28, mde2* or *mde7* by *ago1* or *dcr1* mutations [9]. Furthermore, a recent report identifying Ago1-associated small RNAs by high-throughput sequencing did not find any small RNAs derived from *spo4^+^*, *spo6^+^*, *mug28^+^* or *crp79^+^* and did not show enrichment for the mid-meiotic genes that have antisense transcripts [33].

The RNAi pathway is essential for heterochromatin formation and gene silencing at centromeres, but is dispensable at other heterochromatic loci such as telomeres or the silent mating-type loci (reviewed in [34]). Possibly antisense transcripts could cause heterochromatin formation on mid-meiotic genes independently of the RNAi pathway. This would predict an enrichment of heterochromatin landmarks at these loci. Cam et al. published a comprehensive map of heterochromatin landmarks, including H3K9me and its interacting chromodomain protein Swi6, using ChIP-on-chip of vegetative cells [35]. They observed that H3K9me and Swi6 associate mainly with major heterochromatic loci including centromeres, subtelomeres, the *mat* locus and ribosomal DNA repeats. They also detected a few heterochromatic ‘islands’ corresponded to meiotically-induced genes. However, none of these heterochromatic ‘islands’ correlates with antisense-producing mid-meiotic genes. We concluded that the antisense-mediated repression of mid-meiotic genes is largely independent of heterochromatin formation.

### Meiotic antisense RNAs generate an artefactual “unspliced” signal in some splicing assays

Many intron-containing meiotic genes have been reported to undergoing “meiosis-specific splicing” [16]–[18]. That is, it is thought that these genes are transcribed in vegetative cells but not spliced, and then they become spliced if and when cells enter meiosis. Most of these splicing studies, including one from our laboratory [17] assayed splicing using a standard RT-PCR assay, in which RNA transcripts are incubated with reverse transcriptase and followed by PCR reaction with primers across the intron(s) of interest. A spliced transcript yields a shorter PCR product than an unspliced transcript. However, a weakness in this assay is that it is not strand specific—it will yield a product with an antisense transcript just as efficiently as with a sense transcript, and of course an antisense transcript would not be competent for splicing. Thus, in at least some of these studies, it is likely that in vegetative cells the assay detected the antisense transcript (and of course yielding the longer “unspliced” PCR product), and then in meiotic cells the assay detected the spliced, sense transcript. This would create the illusion that splicing of the sense transcript was specific to meiosis, when in fact what was really happening was that the cells were switching from primarily making the (inherently unspliceable) antisense transcript in vegetative cells, to primarily making the sense transcript in meiotic cells. Another method, high-throughput cDNA sequencing, was also used to assay genome-wide splicing in *S. pombe* [7]. In that report, the authors found a large number of differentially spliced introns, including 254 introns spliced relatively specifically in meiosis, and 478 introns spliced relatively specifically in vegetative cells. However, the sample preparation for sequencing involved PCR amplification in a way that loses strand specificity, and so again, the “unspliced” transcripts could have been either genuinely unspliced sense transcripts, or inherently unspliceable antisense transcripts.

**Figure 5:**
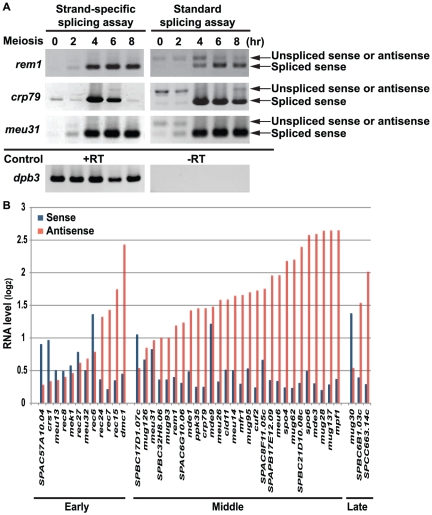
Most “splicing regulated” genes have abundant antisense RNA in vegetative cells. (A) Left: strand-specific splicing assay. Right: standard (non-strand specific) splicing assay. Same RNA samples were used in both assays. Three middle meiotic genes, *rem1, crp79* and *meu31*, were analyzed. The *dpb3^+^* control (+RT) indicates equal loading and the –RT control (minus reverse transcriptase) indicates that samples were not contaminated with genomic DNA. The standard splicing assay shows the unspliced or antisense transcript for all three genes in vegetative or early meiotic cells (2 and 4 hr), while there was no unspliced transcript detected in the same RNA samples using the strand specific splicing assay that only detects sense transcript. More examples can be found at [9]. (B) All genes that were identified as having meiosis-specific splicing are shown here ([16]–[19] and our unpublished data). Genes were separated into three groups, early, middle and late, according to their expression time [26]. Each gene had two values, one for sense RNA (blue bar) and one for antisense RNA (red bar). The values were calculated using average probe intensity on vegetative data. Most middle meiotic genes had much higher antisense RNA level than sense RNA level in vegetative cells. For these genes, the splicing results acquired from the non-strand specific splicing assay were significantly influenced by the presence of antisense RNAs.

*Spo6^+^* is one of the genes with “meiosis specific splicing” that was seen in both these kinds of studies [7], [17], [18]. *spo6^+^* was previously observed to have a vegetative antisense transcript [31]. We and collaborators confirmed this result by strand-specific tiling array (this paper), and also by strand-specific sequencing and strand-specific Northern blot [9]. That is, in vegetative cells, there is far more antisense transcript than sense transcript for *spo6^+^* and also for many other mid-meiotic genes. Here, we developed a strand specific splicing assay, and compared it with the standard non-strand specific splicing assay on *rem1^+^*, *crp79^+^*and *meu31^+^*. For these genes, no “unspliced signal” can be detected with the strand specific assay (Figure 5A). In fact, the sense transcript is in some cases virtually undetectable in vegetative cells. Thus it may be that the strong “unspliced signal” for *spo6^+^* in vegetative cells in the standard assay arises from the antisense transcript, not the (virtually undetectable) sense transcript.

To see if meiosis-specific splicing exists or not, we examined the sense and antisense RNA levels in vegetative cells for all meiotic genes that have been shown, using RT-PCR, to have meiosis-specific splicing [16]–[18]. For most such mid-meiotic genes, we find that the antisense transcript predominates in vegetative cells (Figure 5B). For these genes, the “unspliced” signal in the non-strand specific splicing assay probably reflects antisense RNAs. We conclude that there is currently no good evidence for meiosis-specific splicing of mid-meiotic genes. In marked contrast, the situation is different for the early meiotic genes, many of which are regulated post-transcriptionally by Mmi1 [19], [36]. Most of these early meiotic genes have larger ratios of sense to antisense transcripts in vegetative cells (Figure 5B). Using the strand-specific splicing assay, we have confirmed meiosis-specific splicing for four early meiotic genes, *crs1^+^, rec8^+^, mek1^+^* and *meu13^+^*, and examined the mechanism of meiosis-specific splicing, which seems to depend on Mmi1 [19], [37].

### Fkh2 suppresses sense transcription of mid-meiotic genes in vegetative cells

The mid-meiotic genes are induced by Mei4, a forkhead transcription factor [27]. There are four genes for forkhead transcription factors in *S. pombe*: *mei4^+^* is expressed only in meiosis, while *fkh2^+^*, *sep1^+^* and *fhl1^+^* are expressed in vegetative cells. The core DNA binding motif (GTAAAYA) for forkhead transcription factors is well conserved in fission yeast [38], [39] and likely across species [40]–[42]. Since the core DNA binding motif is similar for many different forkhead transcription factors, an issue arises as to the specificity of certain forkhead factors for certain genes. Part of the answer is that forkhead DNA binding motifs are sometimes found adjacent to motifs for other transcription factors, and these other factors may interact at the protein level only with specific forkhead factors [43].

Of the four *S. pomb*e forkhead transcription factors, *fhl1^+^* is most similar to *S. cerevisiae FHL1*, a gene that regulates ribosomal proteins [44], [45]. Mei4 induces genes that are expressed at meiotic M phase [27]. Sep1 and Fkh2 seem to function together to regulate expression of genes for mitotic M phase [28], [34]. Some forkhead-regulated genes are shared between mitotic and meiotic M phase (like the purple-colored genes in Figure 2), but there are many meiotic M phase genes that are specifically expressed in meiosis (like the red-colored genes). Both categories of genes (i.e., both purple and red) are induced in meiosis by Mei4 [22], but only the purple genes are induced in a vegetative mitosis by Sep1 and Fkh2 [39], [46], but possibly from the same forkhead DNA binding motifs as used by Mei4.

A study on the relationship between transcription factors and splicing efficiency incidentally shed light on the relationship between the vegetative and meiotic forkhead factors [18]. In this report, Moldon et al. found that deletion of *fkh2*, but not *sep1* or *fhl1*, allows splicing of 11 mid-meiotic genes in vegetative cells. We have now shown (Figure 5 and [9]) that the lack of splicing of these transcripts in vegetative cells is because the transcripts in question are probably antisense transcripts. Thus the splicing observed in the *fkh2* mutants may imply that sense transcripts are now being expressed, and constitutively spliced, in *fkh2D* vegetative cells.

**Figure 6:**
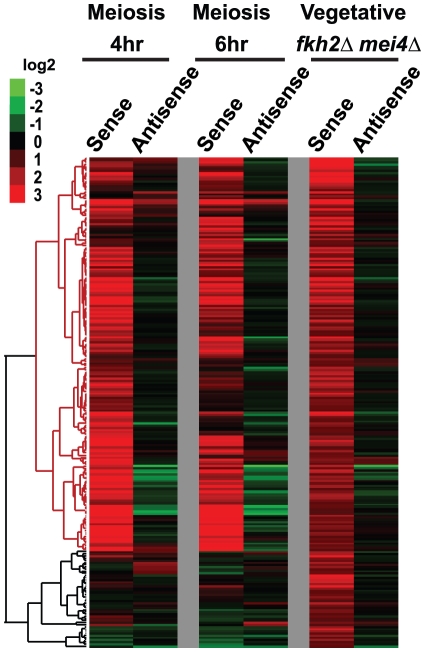
Fkh2 represses expression of mid-meiotic genes. Expression levels for sense and antisense RNAs were calculated using the average probe intensity method. The results presented here are the differences between samples and vegetative cells (e.g., meiosis 4 hr sense level – vegetative sense level). 229 genes that were induced in the *fkh2Δ mei4Δ* strain are shown (cutoff: sense level *fkh2Δ mei4Δ* -WT >1), which include 31 of the 65 “red dot” genes from Figure 2 (p<<10^−10^). Over 75% of these genes are also induced in middle meiosis. Data from meiosis 4 hr, 6 hr and the *fkh2Δ mei4Δ* strain were hierarchically clustered, showing the similarity of regulation of these genes in meiosis and in the mutant. At the bottom of the figure is a sub-cluster of 45 genes induced in the mutant, but not in meiosis; 8 of these genes have no associated GO term, and 18 of these genes respond to stress (p = 0.0006). Red is induced; green is repressed.

To see if this idea is correct, we generated tiling array data using a *fkh2Δ mei4Δ* mutant strain. (The strain was made a *mei4Δ* mutant, because *mei4* has two forkhead binding motifs in its promoter [47]. If indeed Fkh2 represses meiotic genes that have forkhead motifs, then *mei4* is likely to be one of the genes influenced by Fkh2, and would be expressed vegetativly in the *fkh2Δ* strain, inducing meiotic genes and confounding our results). Tiling array analysis shows 229 genes with increased sense RNA level in the *fkh2Δ mei4Δ* mutant (cutoff: *fkh2Δ mei4Δ* -WT >1, Table S5. The key gene studied in Moldon et al., *rem1*, is also induced, but does not pass the cut-off.). Many of these genes were also induced during mid-meiosis (Figure 6). For example, *crp79^+^*, which encodes a meiosis-specific RNA binding protein [29], showed no sense RNA in wild-type vegetative cells, but does show sense RNA expression in the *fkh2Δ mei4Δ* strain (Figure 4B, *crp79^+^*). Overall, the 229 genes up-regulated in the *fkh2Δ* strain included 31 of the 65 “red” genes from Figure 2A (p<10^−10^). This supports our hypothesis that *fkh2^+^* represses sense transcription of mid-meiotic genes. We note that the Fkh2 factor of *S. cerevisiae* also functions as a repressor until Ndd1 and Cdc5 and CDK phosphorylation turn it into an activator in mid-mitosis [48]–[50].

Having found that both disruption of antisense RNA and deletion of *fkh2* allow some sense transcription of mid-meiotic genes, we wondered if the two mechanisms work together to achieve the maximum repression on the same gene. To test this, we generated strains carrying both an antisense KO allele and a *fkh2Δ* allele and assayed the sense and antisense RNA levels. For all three genes tested, the sense RNA level was higher in the double mutant compared to either antisense KO or *fkh2Δ* alone (Figure 3C and Figure S4, last lane). We conclude that antisense RNA and Fkh2 work in concert, but likely through independent mechanisms, to repress mid-meiotic genes in vegetative cells.

### New antisense RNAs appear during meiosis

**Figure 7:**
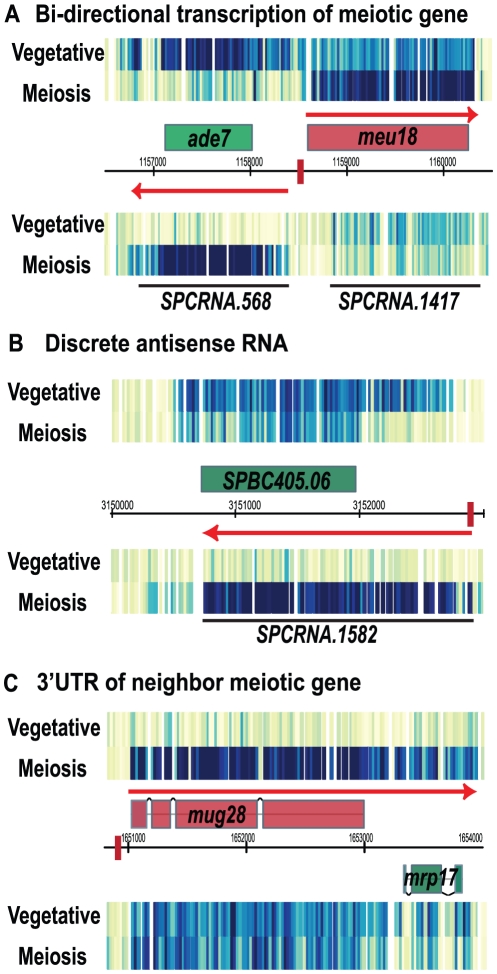
New antisense RNAs appear during meiosis. Genes that have antisense RNA in meiosis are colored green, meiotic genes are colored red and predicted forkhead binding sites are shown as a red box. Red arrows illustrate transcripts that are induced in meiosis (6 hr). The consensus forkhead-binding motif (GTAAAYA) was used to predict forkhead-binding sites. All three examples shown here are new antisense RNAs associated with a predicted forkhead-binding site. (A) Antisense RNA from bi-directional transcription of a Mei4-responsive gene. (B) Discrete antisense RNA that may be induced by a nearby forkhead binding site. (C) Antisense RNA from the 3′UTR of a meiotic gene.

During meiosis, the antisense RNAs for meiotic genes generally decreased. On the contrary, many new antisense RNAs for non-meiotic genes emerged. We inspected the new antisense RNAs with the highest induction level at 6 hr of meiosis (cutoff: 6 hr/veg >2, 48 genes). The sense and antisense RNA levels for these genes also showed a strong inverse correlation (−0.399), suggesting that meiosis-induced antisense RNA may repress sense transcription. The antisense RNAs in meiosis were generally long RNAs and overlapped the entire ORF of the gene on the sense strand (Figure 7), similar to the antisense RNAs observed in vegetative cells (Figure 1). About 83% (40/48) of the meiotically-induced antisense RNAs originated close to a forkhead motif (Figure 7), and so may have been induced by the meiotic forkhead transcription factor Mei4. Many of these 40 antisense transcripts were associated with meiotic sense RNAs; for instance, there was bi-directional transcription from a forkhead motif (Figure 7A), producing a meiotic sense RNA on one side, and an antisense RNA for the adjacent gene on the other side. A second example shows a case in which a forkhead motif is apparently used solely to generate a meiotic antisense RNA over a non-meiotic gene, possibly repressing the sense transcript in meiosis (Figure 7B).

An example of another novel kind of transcription pattern arising from new antisense transcripts in meiosis is the pair *mug28^+^* and *mrp17^+^*, which encode a meiotic RNA-binding protein and a mitochondrial ribosomal subunit, respectively. In vegetative cells, the 3′UTR of *mrp17^+^*, the mitochondrial gene, is the antisense RNA to *mug28^+^* and represses *mug28^+^* expression (Figure 1B and Figure 7C). When *mug28^+^* is induced in meiosis, the 3′UTR of *mug28^+^* covers *mrp17^+^* (Figure 7C) and possibly represses transcription of *mrp17^+^*. Thus these two genes may be mutually repressive through antisense transcription. Some meiosis-induced anti-sense RNAs could be scored as “unspliced” sense meiotic RNAs (see Figure 7C, *mrp17*) and possibly lead to the conclusion that splicing efficiency of these genes decreases during meiosis [7]. As shown in these examples, Mei4 may play a dual role in meiosis: to activate mid-meiotic genes, and also to repress a subset of non-meiotic genes by antisense transcription.

### Genes for spore wall synthesis have internal bi-directional transcription

**Figure 8:**
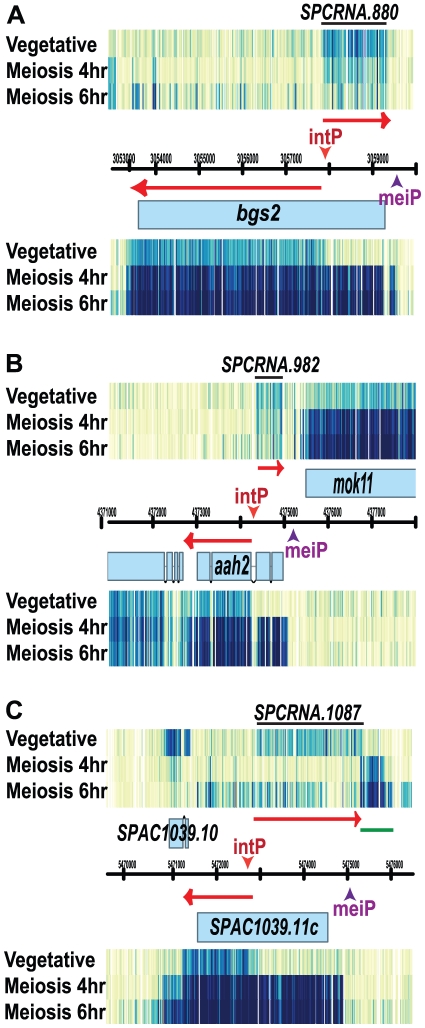
Internal bi-directional transcription. 7 kb window views are shown for three meiotic genes: (A) *bgs2^+^,* (B) *aah2^+^* and (C) *SPAC1039.11c*. For each gene, transcription initiates from an internal promoter that generates a 5′ truncated sense RNA and a divergent non-coding antisense RNA in vegetative cells. The motif (ACGCTC) that might drive the bi-directional transcription is labeled as intP, internal promoter. During meiosis the meiotic promoters, marked as meiP for meiotic promoter, are activated and full-length sense RNAs are made. (B) The meiotic promoter of *aah2^+^* seems to induce bi-directional transcription that generates sense transcription of two meiotic genes, *aah2^+^* and *mok11^+^*. (C) Similarly, the meiotic promoter of SPAC1039.11c seems to induce bidirectional transcription and generates a new non-coding RNA (underlined by a green line) during meiosis.

The characterization of antisense transcripts turned up some unusual patterns of transcriptional control. For example, three genes required for spore wall synthesis, *bgs2^+^* (meiosis-specific 1,3-β-glucan synthase) [51], [52], *aah2^+^* (α-amylase) and SPAC1039.11c (predicted α-glycosidase) shift from promoters in vegetative cells that give non-functional transcripts to different promoters in meiotic cells that give functional transcripts. In vegetative cells, these three genes have bi-directional transcription from a site inside the gene such that two non-overlapping RNAs are produced, one on the sense strand and one on the antisense strand (Figure 8). The sense transcripts are truncated, lacking much of the open reading frame. At mid-meiosis, the functional promoters (marked as meiP, meiotic promoter, in Figure 8) for these meiotic genes are activated and full-length sense transcripts are made. The meiotic promoter of *aah2^+^* seems to activate *aah2^+^* and also, bi-directionally, the adjacent gene *mok11^+^*, an α-1,3-glucan synthase that also functions in spore wall formation (Figure 8B) [53]. This sharing of a meiotic promoter caused *aah2^+^* and *mok11^+^* to be induced at the same time. Notably, the meiotic promoter of SPAC1039.11c also induced bi-directional transcription and generates a new meiotic non-coding RNA (Figure 8C).

We wondered what causes the internal bi-directional transcription. One straightforward possibility is that a DNA motif located inside of these genes is responsible. To test this idea, we retrieved 400 nt of DNA sequence, centered at the 5′ start site of the two divergent transcripts, from each of these three genes. The motif search program MEME [54] found one hexamer motif, ACGCTC, in all three input sequences, with no base substitution (p = 1.33e^−4^) (This motif is marked as intP, internal promoter, in Figure 8). This motif was further analyzed using GOMO (Gene Ontology for MOtifs) [55], which takes the input motif and scores only the promoter region of every gene in the *S. pombe* genome and determines the GO term that associates with the input motif. This search returned a highly significant GO term for ribosomal components (GOMO score = 7.379 e^−10^). Possibly this hexamer motif in vegetative cells recruits an unknown trans-factor(s) for transcribing genes associated with ribosome, but also inhibiting this particular set of meiotic genes by provoking a non-productive transcriptional pattern.

## Discussion

### Repression by antisense transcription

Our tiling-array analysis of gene expression in *S. pombe* found many antisense RNAs, both in vegetative and meiotic cells. In agreement with previous studies [6]–[9], a disproportionate number of the vegetative antisense RNAs were found over mid-meiotic genes. The antisense RNAs over these genes changed in size, or decreased in abundance, when cells went into meiosis and the sense strands were expressed. We believe that the antisense RNAs are causally involved in the vegetative repression of the mid-meiotic genes, because in three cases, when we interfered with the antisense transcription, levels of vegetative sense RNA increased.

There are several mechanisms by which antisense transcription could repress gene expression (for recent review, see [56]). One possibility is that RNA interference (RNAi) is involved. However, deletions of the critical RNAi components *ago1* (argonaute) and *dcr1* (dicer) had no effect on the antisense repression of any of the three genes studied, and a deletion of *rdp1* (RNA-dependent RNA polymerase) affected only *spo6^+^* (Figure 6). In addition, there was no apparent correlation between the Ago1-associated siRNAs identified in a genome wide study [57] and the antisense RNAs identified by us, and there was no apparent correlation between heterochromatic markers [35] and the antisense RNAs. Collectively, these data suggest that the antisense-mediated repression is not occurring primarily through an RNAi mechanism. Since *S. pombe* has a functional RNAi pathway, and since the mid-meiotic genes have significant antisense transcripts, the lack of involvement of the RNAi pathway may be somewhat surprising. However, for RNA interference to occur, the sense RNA has to be transcribed in the first place so that the sense-antisense duplex RNA can be formed and processed by the RNAi machinery. For the four genes studied here (*spo4, spo6, crp79* and *mug28*), sense transcription in vegetative cells was very low (Figure 4), so perhaps there is little or no sense-antisense duplex. In future studies, it would be interesting to see if the RNAi pathway has effects on mid-meiotic genes such as *mug126^+^*, *mde9^+^* and *meu31^+^*, where sense and antisense transcripts are of similar abundance in vegetative cells (Figure 4).

Transcription can influence transcription of a neighboring or overlapping DNA locus by a mechanism called transcription interference. In one kind of transcription interference, a transcribing RNA polymerase sweeps transcriptional activators off of a second promoter (the promoter occlusion model, [58]). In a second kind of transcription interference, a collision between converging RNA polymerases would lead to premature termination (collision model [59]). Transcription interference is used by *S. cerevisiae* in regulating meiotic entry [10], [11]. In a haploid cell, the meiotic gene *IME4* is repressed by antisense transcription, which interferes with sense transcription, whereas in diploid cells, the a1-α2 repressor represses *IME4* antisense and thereby allows *IME4* sense expression [10], [11]. Several features of the antisense RNAs seen here are consistent with the idea that they work by transcription interference. They are polyadenylated, and so probably products of RNA pol II. Importantly, they are typically long and encompass the entire CDS and promoter of the corresponding sense gene. Finally, our terminator-insertion experiment (Figure 3 and Figure S4) presumably does not prevent antisense transcription from initiating, but does prevent it from proceeding over the sense promoter. The effectiveness of these inserted terminators at reducing repression is consistent with a transcription interference model.

A systematic analysis of sense-antisense expression identified that antisense transcription is associated with gene of larger expression variability; i.e. genes that transcribe in an on-off manner [60]. This suggests that antisense transcription confers the switch-like expression pattern for the sense gene, and in our case these vegetatively transcribed antisense RNAs over mid-meiotic genes may keep meiotic genes in the off state. The abundance of antisense RNA for meiotic genes has been recently reported in other budding yeast [5] and in fission yeast species [9] by directly detecting antisense RNAs. Some antisense RNAs are unstable and rapidly degraded [12]. Therefore, it is hard to detect them unless the RNA degradation pathway is compromised [61]. An alternative way to estimate antisense transcription is to look for histone marks that are associated with transcription initiation at the 3′ end of the gene. Using this method, it is estimated that more than 20% of *S. cerivisiae* meiotic genes are transcribed in the antisense direction in vegetative cells [62]. This proportion is larger than that identified by RNA sequencing [5], [63]. This suggests antisense RNAs may play an important role in regulating meiosis.

The vegetative antisense transcripts found over meiotic genes can interfere with splicing assays. Previously, we and others [16]–[18] assayed the “meiosis-specific” splicing of meiotic genes using non-strand specific RT-PCR assays. As shown here, these assays can mistake an (unsplicable) antisense transcript for an unspliced sense transcript. We now believe that many previous reports of meiosis-specific splicing for *S. pombe* mid-meiotic genes are artifacts of this effect. Nevertheless, meiosis specific splicing does occur for some *S. pombe* genes, particularly those important in early meiosis [19], [37]. Such confusion between splicing regulation and antisense transcription may also apply to *S. cerevisiae* [64], and indeed, to any case where a non-strand specific method has been used to assay splicing. The average intensities for sense and antisense of every gene from all samples used in this study are available in Table S3, and these intensities may be useful to researchers in assessing the possibility that an antisense transcript may be interfering with a splicing assay.

### Repression by *Fkh2*

Our finding that the forkhead transcription factor Fkh2 represses meiotic genes in vegetative cells is thought-provoking, because these same genes are induced in meiosis by a different forkhead transcription factor, Mei4. Fkh2, like its homolog in *S. cerevisiae*, is most commonly thought of as a major regulator of mitotic genes.

Fkh2 contains two signature domains. One is the FKH (or “winged helix”) domain for DNA binding; although the exact consensus for *S. pombe* Fkh2 has not been defined, most forkhead transcription factors bind a motif similar to GTAAAYA [38]–[42]. Additional specificity comes from the fact that the forkhead DNA motif is often adjacent to a DNA motif for some other DNA binding protein, and only a specific forkhead transcription factors may have a productive protein-protein interaction with this other protein [43]. The second domain is the FHA (forkhead-associated) domain, which mediates protein-protein interactions with peptides containing phospho-threonine [65]. Fkh2 *of S. cerevisiae* is a repressor when cyclin-dependent kinase activity is low, but becomes an activator after CDK activity climbs, and phosphorylated Ndd1 can interact with Fkh2 through the FHA domain. We have used the word “actipressor” to refer to proteins that are repressors under some conditions and activators under others [66].

Induction of the *S. pombe* mid-meiotic genes, including many of the nuclear division genes regulated in mitosis by Fkh2, requires a different forkhead transcription factor, Mei4. Unlike Fkh2, Mei4 does not have a forkhead-associated domain. Thus regulation of nuclear division genes–some specific for mitosis, some specific for meiosis, and some needed for both—presents some regulatory challenges, since many of the genes are regulated both positively and negatively by forkhead transcription factors, possibly from a common DNA binding motif of approximately GTAAAYA.

One possible scheme for regulation is that the meiosis-specific genes could have an additional DNA binding motif adjacent to the forkhead motif, such that the Fkh2 binds to these genes together with a repressive partner, thus repressing the meiosis-specific genes. In meiosis, Mei4 would replace Fkh2, and the repressive interaction would be lost, thus causing induction of the same set of genes. Obviously more complex variants of this idea could be elaborated. In any case, our results suggest that Fkh2, a forkhead transcription factor, helps (together with antisense transcription) to repress many meiosis-specific genes in vegetative cells, while the same genes can be induced in meiosis by a different forkhead transcription factor, Mei4.

We saw some patterns of transcription that are not easily classified. Many new antisense RNAs appear in meiosis and Mei4 possibly induces them. The present of meiosis specific antisense RNAs was previously identified in budding yeast, and some of these antisense RNAs are likely to be functional [61]. We saw intriguing cases where transcriptional start sites shifted from a vegetative position producing non-functional transcripts to a meiotic position producing functional transcripts (Figure 8), and also cases of possibly mutual antisense repression between pairs of genes with one vegetative member and one meiotic member (Figure 7 A and C).

Finally, it is interesting to note that in meiosis, quite a variety of regulatory mechanisms for gene expression have been noted and may be very common. In addition to classical control through transcription factors and transcription initiation, regulatory mechanisms include a striking modulation of RNA stability [36], [67], [68], meiosis-specific splicing [19], [37], RNA binding proteins, and now, antisense regulation. Some similar observations have also been made in *S. cerevisiae*. It is possible that vegetative and meiotic cells use different palettes of mechanisms for gene regulation.

## Materials and Methods

### Yeast cell culture and meiotic time-course

General *S. pombe* culture methods have been described previously [69]. Strains used in this work are listed in Table S6. Vegetative cells were grown in minimal media (MP biomedicals) with required supplements at 24°C to OD600 = 0.3 to 0.5 upon harvest. A synchronous meiosis was achieved as described [70]. Briefly, a diploid strain homozygous for the *pat1-114* mutation (F277) was grown in EMM2* (without adenine) at 24°C to OD600 = 0.3. Cells were washed with water and resuspended in EMM2* without NH_4_Cl at 24°C for 16 hr to obtain a culture of G1 arrested cells. Cells were shifted to 34°C to inactivate Pat1 and were re-fed with 5 mg/ml NH_4_Cl (time = 0 hour). 2 ml samples were harvested each hour for 8 hours for flow cytometry and DAPI staining (Figure S5) and large samples of 2×10^8^ cells were collected at the same times for RNA isolation.

### RT-PCR based splicing assay

Total RNA was isolated using the RiboPure™-Yeast kit (Ambion). 20 µg of total RNA was treated with 4 U TURBO DNase in 40 µl at 37°C for 1 hr (Ambion). cDNA was synthesized from 4 µg total RNA using SuperScript III reverse transcriptase (Invitrogen) according to manufacturer's instructions and with addition of 50 ng actinomycin D to prevent second strand cDNA synthesis [71]. cDNA for a standard splicing assay was primed with 250 ng random hexamer, while cDNA for a strand specific splicing assay was primed with 100 ng anchored gene-specific primer (gsp). The anchor is a unique sequence at the 5′ end of each gsp and we name this anchor P1. Only the cDNA primed with the anchored gsp would have the P1 sequence, and cDNA primed by fortuitous DNA and RNA fragments that naturally occur in the RNA sample would lack the P1 sequence. cDNA was digested with 0.3 µl 10 mg/ml RNaseA and 1 U RNaseH at 37°C for 30 min to hydrolyze RNA template. To remove unused anchored gsp, cDNA was purified using an absorption spin column that removes oligos smaller than 70 nt (Qiagen, MiniElute). The final volume of cDNA was adjusted to 40 µl and 1 µl was used for the PCR reaction. Forward and reverse primers across the intron were used for the standard splicing assay; the same forward and the P1 reverse primers were used for the strand-specific splicing assay. Therefore, the standard splicing assay detects signals from both sense and antisense RNAs, while the strand-specific assay detects only the cDNA converted from the sense RNA. The PCR reaction was resolved by agarose gel electrophoresis and stained with ethidium bromide. Primer sequences are listed in Table S7.

### Tiling array sample preparation, hybridization and scanning

400 µg of total RNA was mixed with 30 µg dephased oligo(dT) primers (equal molar (dT)_16_-(dA/dG), (dT)_16_-(dC(dA/dG/dC)) in a final volume of 300 µl and incubated for 5 min at 65°C, 2 min on ice and 2 min at room temperature. 90 µl 5× First Strand Buffer, 22.5 µl 0.1 M DTT, 18 µl 10 mM dNTPs and 2 mM dUTP, 4.5 µl 600 µg/µl Actinomycin D (Sigma), 3 µl RNasin, 12 µl Superscript III RT (Invitrogen) and 300 µl water were added to 450 µl. Reverse transcription was performed at 42°C for 16 hr and 2 µl 10 mg/ml RNase A and 10 U RNase H was added to hydrolyze RNA at 37°C for 30 min. Sample was purified using absorption spin column (Qiagen, QIAquick PCR purification). The total recovered cDNA for each sample was between 10–15 µg in 90 ul.

Purified cDNA was fragmented and end labeled as follow. 85 µl cDNA, 10 ul 10× fragmentation buffer (Affymetrix, GeneChip® WT Double-Stranded DNA Terminal Labeling Kit), 2 µl UDG (uracil DNA glycosidase) and 3 µl APE1 (apurinic/apyrimidinic endonuclease) were incubated at 37°C for 1 hr. To stop the fragmentation reaction, sample was heated to 93°C for 10 min and cooled on ice. 93 µl of fragmented cDNA was incubated in a reaction containing 30 µl 5× TdT buffer, 3 µl DNA labeling reagent (Affymertix) and 16 µl H_2_O at 37°C for 1 hr to label the cDNA ends. To stop the labeling reaction, sample was heated to 70°C for 10 min and cooled on ice.

For every sample, tiling array hybridizations were performed in triplicate. 150 µl hybridization cocktail, which contains 5 µg of labeled cDNA, 2.5 µl Control Oligo B2 (Affymetrix), 75 µl 2× hybridization buffer and 10.5 µl DMSO, was prepared for each array cartridge (Affymetrix, S. pombe tiling 1.0). Hybridization cocktail was denatured at 99°C for 5 min followed by slow cooling in an air incubator set at 45°C for 5 min. 130 µl of hybridization cocktail was loaded into the array cartridge and hybridized at 45°C for 16 hr with constant rotating at 60 rpm. Array was washed and stained according to manufacturer instructions (Affymetrix FS450 fluidic station and FS450_0002 protocol). Array was filled with 160 µl Array Holding Buffer and immediately scanned on a GeneChip® Array Scanner (Model 3000-7G). Grids were placed and aligned to raw image files with GeneChip Operating System 1.4 (Affymetrix). The resulting cell level summary files (.cel) were used for analysis.

### Tiling array analysis

*S. pombe* Tiling 1.0 array probe sequences were obtained from Affymetrix. Probes were mapped to Sanger *S. pombe* genome sequence (April 2007 version) using xMAN [72]. Probe intensity files (.cel) that contains the raw intensity were normalized to genomic DNA hybridization to correct probe effects and background correction. Probes that mapped the genome perfectly once were used to correct for probe effects, and a subset of these probes, which mapped outside of the CDS were used for background correction. The normalized data was segmented using the Change Point Segmentation Model. Bioconductor package “tilingArray” [1] was used for these analyses. To calculate the average probe intensity of each segment, the signal intensities of every probe located within a segment were added up and divided by the numbers of probe in the segment. Antisense segments in vegetative cells with average intensity over an arbitrary threshold of 1.0 were defined as antisense RNAs. To demonstrate the level of sense and antisense RNA intensities for every gene (as shown in Figure 2 A and B), probes located within the CDS (from ATG to stop codon) were used to calculate the average intensity for sense and antisense RNAs.

### Strain constructions

The *ura4* terminator (U1) flanked the selectable marker *ura4* as a direct repeat to form the ter-*ura4* cassette (teminator-promoter-*ura4*-terminator). This cassette was cloned. The promoter-*ura4*-terminator sequence was PCR amplified with primers *ura4*-Pro-*Hind*III and *ura4*-Ter-*EcoR*I into pSC-AK (Stratagene) between *Hind*III and *EcoR*I sites; this plasmid was named pSC-*Ura4*. The U1 terminator was PCR amplified with primers *ura4*-Ter-U15′-*BamH*I and *ura4*-Ter-U13′-*HindIII* and cloned in front of the ura4 promoter at *BamH*I and *Hind*III sites of pSC-*ura4*; this plasmid was named pSC-ter*-Ura4.*

To make the *Spo6-*AS-KO1 strain, we further cloned upstream and downstream regions, relative to the insertion site, that would direct recombination flanking the ter-*ura4*. The upstream and downstream regions were PCR amplified with primers c1778.05c-5′F2-*Xba*I/c1778.05c-5′R-*BamH*I and c1778.05c-3′F-*EcoR*I/c1778.05c-5′ R2-*Xho*I, respectively. The upstream and downstream PCR products were sequentially cloned into pSC-ter-*Ura4* between *Xba*I/*BamH*I and *EcoR*I/*Xho*I sites. This plasmid was digested with *Xba*I and *Xho*I and transformed into a WT diploid strain that carries the *ura4-D18* allele. Correct recombination would disrupt SPBC1778.05c, the 3′UTR of which is the source of the *spo6* antisense transcript, and this recombinant would carry a functional *ura4* allele. Tetrad dissection of recombinants recovered from minus uracil plates showed two to two segregation of the Ura^−^ and Ura^+^ phenotypes and all the Ura^+^ colonies (SPBC1778.05c disrupted, *Spo6-*AS-KO1 strains) were smaller than Ura^−^ colonies (data not shown). This suggests that the sequence orphan SPBC1778.05c was responsible for this slow growth phenotype. SPBC1778.05c was PCR amplified with primers c1778.05c-*Xho*I-ATG/c1778.05c-*BamH*I-Stop and cloned into p*Rep41*-XL between *Xho*I and *BamH*I sites; this plasmid was p*Rep41*-c1778.05c. Transformation of p*Rep41*-c1778.05c into *Spo6-*AS-KO1 strains rescued the slow growing phenotype. This *Spo6-*AS-KO1 strain was counter selected using 5-FOA so as to remove *ura4* by recombination between the two direct U1 terminator repeats, leaving a single U1 terminator in the correct place and orientation. This strain was confirmed by Southern blotting and sequencing (data not shown). All experiments with *Spo6-*AS-KO1 strains contained the SPBC1778.05c-complementing plasmid p*Rep41*-c1778.05c. The same strategy was used to generate the *spo4*-AS-KO strain.

To make the *Spo6-*AS-KO2 strain, we cloned different upstream and downstream regions for recombination flanking *ura4^+^* (promoter-*ura4^+^*-terminator). Two-step overlapping PCR was used for this construction. The first PCR involved three fragments: *ura4^+^*, upstream and downstream regions, that were PCR amplified with primers *ura4*-pro/*ura4*-ter, *spo6*-exo3F/*spo6*-150R-*ura4*P and s*po6*-150F-*ura4*T/*spo6*-441R, respectively. The three fragments each overlapped by 30 nt. 10 cycles of PCR with equal molar amounts of the three segments were preformed and followed by another 20 cycles of PCR with the two outer-most primers, *spo6*-exo3F/*spo6*-441R. This PCR product was transformed into a WT haploid cell that carries the *ura4-D18* allele. Colonies recovered from minus uracil plate were sequenced (data not shown). The same strategy was used to generate the *mug28*-AS-KO strain. Primers for generating these AS-KO strains are listed in Table S7.

### Semi-quantitative RT-PCR

Semi-quantitative PCR with α-^32^P-dCTP was used for measuring the sense and antisense RNA levels. Sense cDNA and antisense cDNA were synthesized with anchored gene-specific primers (gsp) that complement the sense or antisense RNA, respectively. The anchor sequence for sense-gsp was P1 and for antisense-gsp was P2. Primers are listed in Table S7. Other cDNA synthesis steps were the same as described above. Each 20 µl PCR mixture contained 1 µl cDNA and 2 µCi α-^32^p-dCTP. 18 cycles of PCR were performed and 5 µl of sample was resolved on a 5% TBE-acrylamide gel. The desiccated gel was imaged using a Phosphor Storage Screen (Molecular Dynamics). Signals were detected and analyzed using the Phosphoimager Storm system (GE) and ImageQuant software (GE).

## Supporting Information

Figure S1 **Most genes with high antisense and low sense level in vegetative cells are induced during meiosis.** The differences of sense transcript level between meiosis 4 hr and vegetative cell (red bar) and between meiosis 6 hr and vegetative cells (green bar) are shown for the 116 genes that have high antisense to sense ratio in vegetative cells. The genes are ranked from left to right of the figure by their sense induction levels in meiosis 6 hr. Only 10 genes out of the 116 do not exhibit an increased sense expression neither at meiosis 4 hr nor at meiosis 6 hr (the genes on the right side of the figure), while the majority of the genes are induced during meiosis.
(EPS)

Figure S2 **Sense and antisense transcription antagonize each other.** Changes in sense expression are anti-correlated with changes in antisense transcript levels. Differences in expression level between the vegetative and meiosis 6 hr samples are shown. Genes previously identified as Mei4 responsive, or mid meiotic genes, shown in red, are generally induced in meiosis. Pearson correlation for all genes is −0.221 and for Mei4 responsive gene is −0.453.
(EPS)

Figure S3 ***spo6^+^*** **antisense changes in meiosis 6 hr.** The antisense strand of *spo6^+^* is transcribed into short RNA fragments in meiosis 6 hr. The regions amplified in the radioactive PCR shown in Figure 3 are marked; green region is for sense RNA and red region is for antisense RNA.
(EPS)

Figure S4 **Disruption of antisense transcription allows** ***spo4^+^*** **and** ***mug28^+^*** **sense transcription in vegetative cells.** (A) Left: illustration of *spo4^+^* antisense disruption strain. The U1 terminator was inserted in the same orientation as *spo4^+^* antisense transcription at the 5′ end of the antisense region (the same strategy as for *spo6-*AS-KO1). Right: RNA from two independent KO transformants was analyzed (KO #1 and KO #2). Antisense RNA decreased and sense RNA increased in the KO strains. Deletion of *fkh2* also allowed a low level of *spo4^+^* sense RNA expression. Sense RNA level became more abundant in the strain with both antisense disrupted and *fkh2Δ.* (B) Left: illustration of *mug28^+^* antisense disruption strain. The *ura4^+^* cassette (promoter-*ura4^+^*-terminator) was inserted between *mug28^+^* and *mrp17^+^* in the same transcription direction as *mug28^+^* (the same strategy as for *spo6-*AS-KO2). The results with *mug28^+^* were very similar to the results with *spo4^+^* except that *mug28^+^* sense transcription was apparent in the *fkh2Δ* mutant. *adh1^+^* is included as internal loading control, and *adh1^+^* (-RT) indicates no genomic DNA contamination. Two or more isolates for each strain were assayed. This figure shows the representative result.
(EPS)

Figure S5 **Synchronized meiosis.** Diploid *pat1-114/pat1-114* (F277) was induced to enter meiosis synchronized. Cells were stained with DAPI to visualize chromosome and 200 cells from each time point were counted. Meiotic DNA synthesis was between 2–4 hr after induction, first meiotic division was around 4.5–5.5 hr and second meiotic division was around 5.5–6.5 hr.
(EPS)

Table S1 **Summary of five genome-wide studies of antisense RNAs in log-phase vegetatively grown** ***S. pombe*** **cells.**
(DOC)

Table S2 **Strandness, boundaries, intensity and category of transcripts in vegetative cells.**
(XLS)

Table S3 **Sense to antisense ratio of all genes in vegetative, meiotic,** ***fkh2Δmei4Δ*** **and** ***rdp1Δ*** **cells.**
(XLS)

Table S4 **List of the 116 genes with higher antisense to sense ratio.**
(XLS)

Table S5 **Genes that are increased more than 2 fold in the** ***fkh2Δmei4Δ*** **strain.**
(XLS)

Table S6 **Strain list.**
(DOC)

Table S7 **Primer list.**
(DOC)
