# Supplementary material for: Repression of Meiotic Genes by Antisense Transcription and by Fkh2 Transcription Factor in Schizosaccharomyces pombe
Source: PLoS One. 2012 Jan 6;7(1):e29917. doi: 10.1371/journal.pone.0029917 (PMC3253116; doi:10.1371/journal.pone.0029917)
Supplement: Table S1 — Summary of five genome-wide studies of antisense RNAs in log-phase vegetatively grown S. pombe cells. (DOC) [file pone.0029917.s006.doc]

**Table S1.** Summary of five genome-wide studies of antisense RNAs in log-phase vegetatively grown *S. pombe* cells

| **Publication** | **Platform** | **Sample preparation^1^** | **Strand specificity^2^** | **Threshold^3^** | **Number of antisense RNAs** | **GO term for meiosis** |
| --- | --- | --- | --- | --- | --- | --- |
| Wilhelm et al. 2008 | Affymetrix | total | weak | Manual inspection | 37 | N/A, but mentioned in the text |
|  | Sequencing | polyA | weak |  |  |  |
| Dutrow et al. 2008 | Affymetrix | polyA and total | good | 3.5 fold above base line, >180nt | 2483 | N/A |
| Ni et al. 2010 | DeLi-seq | polyA | excellent | q<0.01 | 2409^3^ | 5.1×10^-11^ |
| Rhind et al. 2011 | Sequencing | polyA | excellent |  | 648 | 10^-10^ |
| This study | Affymetrix | polyA | good | Intensity >1, segment > 150nt | 1540 | 9.1×10^-11^ |

^1^ Vegetative sample is called normal, log-phase and MM (minimum medium) in these publications. PolyA: sample was enriched for polyadenylated RNA; Total: total RNA.

^2^ Base on both sample preparation and platform used, we subjectively assign the strand specificity.

^3^ See original papers for detail.

^4^This is the combined number from vegetative and heat-shock samples.
