# Supplementary material for: Repression of Meiotic Genes by Antisense Transcription and by Fkh2 Transcription Factor in Schizosaccharomyces pombe
Source: PLoS One. 2012 Jan 6;7(1):e29917. doi: 10.1371/journal.pone.0029917 (PMC3253116; doi:10.1371/journal.pone.0029917)
Supplement: Table S6 — Strain list. (DOC) [file pone.0029917.s011.doc]

**Table S6.** Strains used in this work. Strain names in parenthesis are the original name from the requested laboratory or from Yeast Genetic Resource Center.

| **Strain Name** | **Genotype** | **Reference/Source** | |
| --- | --- | --- | --- |
| JLP988 | *h^-^  ade6-M216 leu1-32 his7-366 ura4-D18* | Lab stock |  |
| F277 (FY16057) | *h^+^/h^+^ pat1-114/pat1-114 ade6-M210/ade6-M216* | YGRC |  |
| JLP1486 | *h^-^  spo6-*AS-KO1 *ade6-M216 leu1-32 his7-366 ura4-D18* | This work |  |
| JLP1673 | *h^-^  spo6-*AS-KO2 *ade6-M216 leu1-32 his7-366 ura4-D18* | This work |  |
| JLP1675 | *h^-^  spo4*-AS-KO *ade6-M216 leu1-32 his7-366 ura4-D18* | This work |  |
| JLP1677 | *h^-^  mug28*-AS-KO *ade6-M216 leu1-32 his7-366 ura4-D18* | This work |  |
| JLP1674 | *h^-^ spo6-*AS-KO2 *fkh2::ura4^+^  ade6-M216 leu1-32 his7-366 ura4-D18* | This work |  |
| JLP1676 | *h^-^ spo4*-AS-KO *fkh2::ura4^+^  ade6-M216 leu1-32 his7-366 ura4-D18* | This work |  |
| JLP1678 | *h^-^ mug28*-AS-KO *fkh2::ura4^+^  ade6-M216 leu1-32 his7-366 ura4-D18* | This work |  |
| JLP1501 | *h^-^ fkh2::ura4^+^ ade6-M210 leu1-32 ura4-D18* | This work |  |
| JLP1500 | *h^-^ fkh2::ura4^+^mei4::ura4^+^ ade6-M210 leu1-32 ura4-D18* | This work |  |
| F322 (yFS316) | *h^+^ ago1::kanMX leu1-32 ura4^-^ ade6-210 adh1:gfp* | [1] |  |
| F323 (yFS317) | *h^−^ rdp1::kanMX leu1-32 ura4^-^ ade6-210 adh1:gfp* | [1] |  |
| F324 (yFS318) | *h^−^ dcr1::kanMX leu1-32 ura4^-^ adh1:gfp* | [1] |  |

1. Sigova A, Rhind N, Zamore PD (2004) A single Argonaute protein mediates both transcriptional and posttranscriptional silencing in Schizosaccharomyces pombe. Genes Dev 18: 2359-2367.
