# Supplementary material for: Repression of Meiotic Genes by Antisense Transcription and by Fkh2 Transcription Factor in Schizosaccharomyces pombe
Source: PLoS One. 2012 Jan 6;7(1):e29917. doi: 10.1371/journal.pone.0029917 (PMC3253116; doi:10.1371/journal.pone.0029917)
Supplement: Table S7 — Primer list. (DOC) [file pone.0029917.s012.doc]

**Table S7.** Primer list

| **AS-KO strains construction** | | | | | | | |
| --- | --- | --- | --- | --- | --- | --- | --- |
| pSC-*ura4* | | | *ura4*_Pro_*Hind*III | | | CCCAAGCTTagctacaaatcccactggcta | |
|  |  |  | *ura4*_Ter_*EcoR*I | | | GGAATTCgtgatattgacgaaactttttgac | |
| pSC-ter*-ura4* | | | *ura4*-Ter-U15’_*BamH*I | | | CGGGATCCgggaataaaaagtaatttgctatag | |
|  |  |  | *ura4*-Ter-U13’_ *HindIII* | | | CCCAAGCTTgtgatattgacgaaactttttgac | |
| *spo6-*AS-KO1 | | | c1778.05c_5'F2_*Xba*I | | | GCTCTAGAtcagataccaaactgcgtag | |
|  |  |  | c1778.05c_5'R_  *BamH*I | | | CGGGATCCatcaattgaaatggcggcta | |
|  |  |  | c1778.05c_3'_F_  *EcoR*I | | | GGAATTCcccattattattagggattgttgg | |
|  |  |  | c1778.05c_3'_R2_  *Xho*I | | | CCGCTCGAGgtgtgttggtgcgtcaaatc | |
| p*Rep41*-c1778.05c | | | c1778.05c-*Xho*I-ATG | | | CCGCTCGAGatgtattcactttatactgcaacca | |
|  |  |  | c1778.05c-*BamH*I-Stop | | | CGGGATCCTTAGACTGGTTTTCCAAGCGTG | |
| *spo6-*AS-KO2 | | | *ura4*_Pro | | | agctacaaatcccactggcta | |
|  |  |  | *ura4*_Ter | | | gtgatattgacgaaactttttgac | |
|  |  |  | *spo6*-exo3F | | | acctaatgccttcgatgcag | |
|  |  |  | *spo6*-150R-*ura4*P | | | gcatacatatagccagtgggatttgtagctACATCATTATATTGTTAATTTTCTCTTC | |
|  |  |  | *spo6*-150F-*ura4*T | | | ttagatgtcaaaaagtttcgtcaatatcacgttgtagcattcttttcttaaac | |
|  |  |  | *spo6*-441R | | | ttgagtttgaaagggggaaa | |
| *spo4*-AS-KO | | | *spo4*-KO5’F-*Xba*I | | | GCTCTAGAaacgcacgatagcacctttt | |
|  |  |  | *spo4*-KO5’R-*BamH*I | | | CGGGATCCcggtcaaatactaagtacag | |
|  |  |  | *spo4*-KO3’F-*Sal*I | | | ACGCGTCGACattcgcttttctcacgtgct | |
|  |  |  | *spo4*-KO3’R-*Xho*I | | | CCGCTCGAGagccattgacttgttggaca | |
| *mug28*-AS-KO | | | *mug28*-KO5’F | | | gcatacatatagccagtgggatttgtagcttcaaccgttgttaacgactcc | |
|  |  |  | *mug28*-KO5’R-*ura4*P | | | cgccctctctaacaattcca | |
|  |  |  | *mug28*-KO3’F-*ura4*T | | | ttagatgtcaaaaagtttcgtcaatatcacggacagctctgcgaatattttt | |
|  |  |  | *mug28*-KO3’R | | | gcggtaatacctcgtttttgc | |
| **Splicing assay** | | | | | | | |
| For each gene, primers are listed in the following order: (1) primer for strand–specific cDNA synthesis, (2) forward primer for both strand-specific and standard splicing assays and (3) reverse primer for standard splicing assay. P1 primer was used as reverse primer for strand-specific splicing assay. | | | | | | | |
| *rem1* | | SSS-P1-*rem1* | | | GGTCACCTTGATCTGAAGCatgtatgcacccccacaatc | | |
|  |  | *rem1*-6LG2 | | | CAGCAGATGAATCGGTGTCT | | |
|  |  | *rem1*-6RG2 | | | TCCCTCCTTTTTCTCTTCCC | | |
| *crp79* | | SSS-P1-*crp79* | | | GGTCACCTTGATCTGAAGCGGCTGGATGATTTTGCTGAT | | |
|  |  | *crp79*-exo1F | | | GTCCCCGGACAGTATGAAGA | | |
|  |  | *crp79*-exo4R | | | GGCTGGATGATTTTGCTGAT | | |
| *meu31* | | SSS-P1-*meu31* | | | GGTCACCTTGATCTGAAGCAGAAGGCATCAATCGTGGAC | | |
|  |  | *meu31-*3LC4 | | | GCATAAGTGAAATCGGCAAA | | |
|  |  | *meu31-*3RC4 | | | GAAGAAGGCATCAATCGTGG | | |
| *dpb3* | | *dpb3*-5’F | | | gcagatttcctgttgctcgt | | |
|  |  | *dpb3*-3’R | | | acgcggaagaggcttcacta | | |
| P1 | |  | | | GGTCACCTTGATCTGAAGC | | |
| **Semi-quantitative PCR** | | | | | | | |
| Primers for sense cDNA synthesis (SSS-P1-*gene*) and forward primer for sense strands PCR are list above, except for *spo6*. Primers are listed in the following order: for sense cDNA synthesis (SSS-P1-*gene*), forward primer for sense strands PCR, primer for antisense cDNA synthesis (SSS-P2-*gene*) and forward primer for antisense strand PCR. P1 and P2 primer were used as reverse PCR primer for sense and antisense strand, respectively. | | | | | | | |
| *spo6* | SSS-P1-*spo6* | | | | GTCACCTTGATCTGAAGCCGTCGGATTAGCAAAAACAAA | | |
|  | *spo6-*exo3F | | | | ACCTAATGCCTTCGATGCAG | | |
|  | SSS-P2-*spo6* | | | | GCTTCAGATCAAGGTGACCacctaatgccttcgatgcag | | |
|  | *spo6-*exo3R | | | | agagattgcaagcacgaagc | | |
| *spo4* | SSS-P1-*spo4* | | | | GGTCACCTTGATCTGAAGCGCTGTTTTGGCCTTTACTCG | | |
|  | *spo4-*A10L | | | | CCTCCAGAGGGTTACTTGCTAC | | |
|  | SSS-P2-*spo4* | | | | GCTTCAGATCAAGGTGACCagccattgacttgttggaca | | |
|  | *spo4-*exo3R | | | | GCTGTTTTGGCCTTTACTCG | | |
| *mug28* | SSS-P1-*mug28* | | | | GGTCACCTTGATCTGAAGCAAATGGATTTGGCAAAGCAG | | |
|  | *mug28*-exo1F | | | | GCCAAAGCTCAGATCTTCA | | |
|  | SSS-P2-*mug28* | | | | GCTTCAGATCAAGGTGACCtgaaaaatttcacgccattg | | |
|  | *mug28*-exo4R | | | | AAATGGATTTGGCAAAGCAG | | |
| *crp79* | SSS-P2- *crp79* | | | | GCTTCAGATCAAGGTGACCagcttatggcctttcccatc | | |
|  | *crp79*-exo4R | | | | GGCTGGATGATTTTGCTGAT | | |
|  | SSS-P1-*crp79* | | | | GGTCACCTTGATCTGAAGCGGCTGGATGATTTTGCTGAT | | |
|  | *crp79*-exo1F | | | | GTCCCCGGACAGTATGAAGA | | |
| P2 |  | | | | GCTTCAGATCAAGGTGACC | | |
| SSS-P2-*spo6* | | | | GCTTCAGATCAAGGTGACCacctaatgccttcgatgcag | | |  |
